# 

**Published:** 1853-04

**Authors:** 


### CONTENTS OF THE APRIL NUMBER.

### ORIGINAL COMMUNICATIONS.

Page

ART. I.—Contributions to the Chemical and Physiological History of Diabetes Mellitus. By J. Bryant Smith, M. D., of New York City, - . - - - - - - - - 657

ART. II.—Pneumonia. By Smelfungus, - - - - - - - - - - - - - - 662

ART. III.—Four Cases of Glossitis. By T. D. Strong, M. D., Westfield, N. Y., - - - - 667

ART. IV.—Report of the Obstetric Committee on Anæsthesia in Midwifery and the Speculum Uteri. By Henry Miller, M. D., of Louisville, Ky. Reprinted for private distribution, from the Transactions of the Kentucky State Medical Society, 1852. - - - - - 670

### ECLECTIC DEPARTMENT.

The Country Doctor. - - - - - - - - - - - - - - - 683

Case of Portal Phlebitis, - - - - - - - - - - - - - - - - 9[illegible]7

Observations on the Treatment of some forms of Obstruction of the Bowels, - - - - 688

On Bright's Disease, - - - - - - - - - - - - - - - - - - 692

Dr. Wood's Treatment of Scarlet Fever, - - - - - - - - - - - - 695

Prospectus of Massie's Eclectic Southern Practice, - - - - - - - - - 696

On the Use of P[illegible]atives in the Treatment of Bilious Fevers, and other Bilious Affections of the South and West, - - - - - - - - - - - - - - - 697

Wry Neck cured without cutting, - - - - - - - - - - - - - - 701

Bite of a Copperhead—"Trigonocephalus Contortix"—treated with Whisky, - - - - 703

The Medical Profession and American Slavery, - - - - - - - - - - - 704

### EDITORIAL DEPARTMENT.

New York State Medical Society, - - - - - - - - - - - - - - 705

Anatomical Bill, - - - - - - - - - - - - - - - - - - - 714

Medical Department of the University of Buffalo, - - - - - - - - - - 715

A Discourse on the Life, Character and Services of Daniel Drake, M. D., - - - - - 717

Marshall Hall, M. D.; - - - - - - - - - - - - - - - - - - 718

Meeting of the American Med. Association, - - - - - - - - - - - - 718

Posthumous volume of Dr. Drake's treatise on the diseases, etc., of the interior valley, - - 719

Decease of Prof. Horner, - - - - - - - - - - - - - - - - 719

Obituary, - - - - - - - - - - - - - - - - - - - - - 719

Death of Dr. Pereira, - - - - - - - - - - - - - - - - - - 720

